# Partnering to power progress towards a paradigm shift: Response to Riggare et al

**DOI:** 10.1111/hex.13126

**Published:** 2020-09-16

**Authors:** Lisa Cone, Megan Feeney, Christiana Evers, Danielle Agpalo, Jori Fleisher, Karlin Schroeder

**Affiliations:** ^1^ Parkinson’s Foundation Wheat Ridge Colorado USA; ^2^ Parkinson’s Foundation New York New York USA; ^3^ Department of Neurological Sciences Section of Movement Disorders Rush University Medical Center Chicago Illinois USA; ^4^ Lewy Body Dementia Association Research Center of Excellence Section of Movement Disorders Rush University Medical Center Chicago Illinois USA; ^5^ CurePSP Center of Care Section of Movement Disorders Rush University Medical Center Chicago Illinois USA

I’m Lisa Cone, a 56‐year‐old person diagnosed with Parkinson's disease in 2008 and a patient advocate. While I consider myself an active advocate, I have no blog, personal webpage or social media platform from which I connect with engagement opportunities. What I have is my personal experiences as a patient, knowledge of the US healthcare system gleaned from my executive‐level professional experience, time (thanks to a forced early departure from the workplace) and a passion to improve my life and the lives of others who live with Parkinson's disease.

In 2014, I was on the first patient advisory board for a patient‐focused organization. My fellow patient board members were, like me, demanding to be more involved in their own care, but we were running into roadblocks. One big area of focus was the resistance to patients as participants in the process of drug development for our various health conditions. To better understand these roadblocks, I participated in interviews with leaders in the drug development industry. These interviews culminated in an article I co‐authored in 2016 by Value in Healthcare titled, ‘*Increasing Patient Participation in Drug Development*’.^1^

From that article, interviewees reported a number of barriers and challenges to expanding patient involvement in drug development, one of which included methodological concerns, specifically ‘the lack of uniform, repeatable and scientifically rigorous methods for involving patients’.^1^ We are still hearing this today.

So, when I had the opportunity to address this resistance and provide an answer to the demand for scientifically rigorous methods for involving patients, I jumped at it once again. Partnering with staff at the Parkinson's Foundation and Jori Fleisher, MD, we authored the recently published viewpoint article, ‘*Utilizing patient advocates in Parkinson's Disease: A proposed framework for patient engagement and the modern metrics that can determine its success*’.^2^ We provided uniform, repeatable steps as requested by researchers across academic, government, pharmaceutical and patient advocacy organizations who have sought our assistance to include patients as partners in their work.

In response to Riggare et al,^3^ myself and my co‐authors agree with many of the points raised.

Yes, it would be absurd for patients not to be included in the work of building a methodology and metrics. That is why I co‐authored this paper and why the methodology developed by the Parkinson's Foundation is built on lessons learned from over 20 years of partnering with the Parkinson's community, amongst other people who are active in patient engagement in research.

Yes, Parkinson's disease has its share of active patients, many of whom I’ve had the pleasure of collaborating with as part of the Parkinson's Foundation's network of 350 Research Advocates. My co‐authors and I are so glad all of these global voices are in this space, moving patient engagement forward. I have learned through this work that people living with Parkinson's aren't our only allies—many researchers, advocacy organizations and other partners are working to accomplish this same goal. We have the opportunity to move patient engagement forward successfully through collaboration.

Yes, patient engagement should always be mandatory, and it is, indeed, problematic, that it is not. Yes, patients are, and rightfully should be, the experts in their individual lived experience with the disease, and thus critical as advocates in research. However, unfortunately, the ethical argument alone for patient engagement has not been enough to create the paradigm shift we all want. This is evidenced by organizations like the Patient‐Centered Outcomes Research Institute or guidances such as the US Food and Drug Administration’s Patient‐Focused Drug Development requesting that patients, researchers, pharmaceutical and advocacy organizations assist in the creation of measurable approaches to patient engagement to further promote engagement in future research. Their goal of measurement is not to justify patient engagement as a commodity, but instead to improve the process to maximize impact on research projects. These initiatives would not exist if the patient engagement field didn't agree that there is a need to generate evidence for how patient engagement improves and expedites research. It takes partnerships across disciplines and healthcare to pair state‐of‐the‐science knowledge, lived experiences, and expertise in education, advocacy, industry, public health and philanthropy to create change. It takes a framework to guide expectations, roles and priorities to power progress.

Yes, patient engagement should never be compartmentalized. The approach to patient engagement included in our paper reinforces that engagement should occur at all stages of research. This is clear in our industry example, a real‐world model, which served to highlight a case study, not to represent the entire realm of possible patient engagement projects. The patient advocates involved successfully changed study protocol, resulting in a complete re‐start of the study months after it originally launched. This project was so successful that we had many other patient engagement partnerships with the same company, including one that I engaged in. I took this example, along with others, and shared it with a couple hundred people in another division of the company to educate them about how best to collaborate with people in their disease communities.

I have worked with multiple patient‐focused organizations and with dozens of advocates of all backgrounds and perspectives. Most of these advocates and organizations welcome collegial fact‐based dialogue as we try to solve the same problems. We do too. We encourage all readers to read our paper. Ask questions. Give feedback.

And we encourage you to consider: just as people living with a condition are first and foremost people (never ‘just patients’), with all the richness of life experiences and knowledge that we all have, similarly, ‘academics’ and ‘charity officers’ are more than just people doing a job. Many of them are driven to this field by firsthand experience with health conditions and motivated to stay in these fields and advocate alongside the community out of deep emotional connections. This work is important to all of us. We all have too much on the line.

## CONFLICTS OF INTEREST

Jori Fleisher has received research support from NIH/NINDS, CurePSP, Biogen, Joyce DeMoose & George Harvey (private philanthropy). Jori Fleisher is a consultant to UCB. Jori Fleisher has received royalties from UpToDate. No conflicts of interest are reported for all other authors.
